# Correction to HA15 inhibits binding immunoglobulin protein and enhances the efficacy of radiation therapy in esophageal squamous cell carcinoma

**DOI:** 10.1111/cas.15923

**Published:** 2023-08-18

[Luo H, Wang L, Zhang D, Sun Y, Wang S, Song S, Ge H. HA15 inhibits binding immunoglobulin protein and enhances the efficacy of radiation therapy in esophageal squamous cell carcinoma. Cancer Sci. 2023 Apr;114(4):1697–1709. doi: 10.1111/cas.15712. Epub 2023 Jan 11. PMID: 36582172; PMCID: PMC10067410.]

The authors found that it was not rigorous enough to use HSP90 as loading control for Figure 4C and Figure 5G. Therefore, they performed the experiment in Figure 4C and Figure 5G again and used β‐actin as housekeeping proteins.

The corrected Figures 4 and 5 are shown here:

**FIGURE 4:**
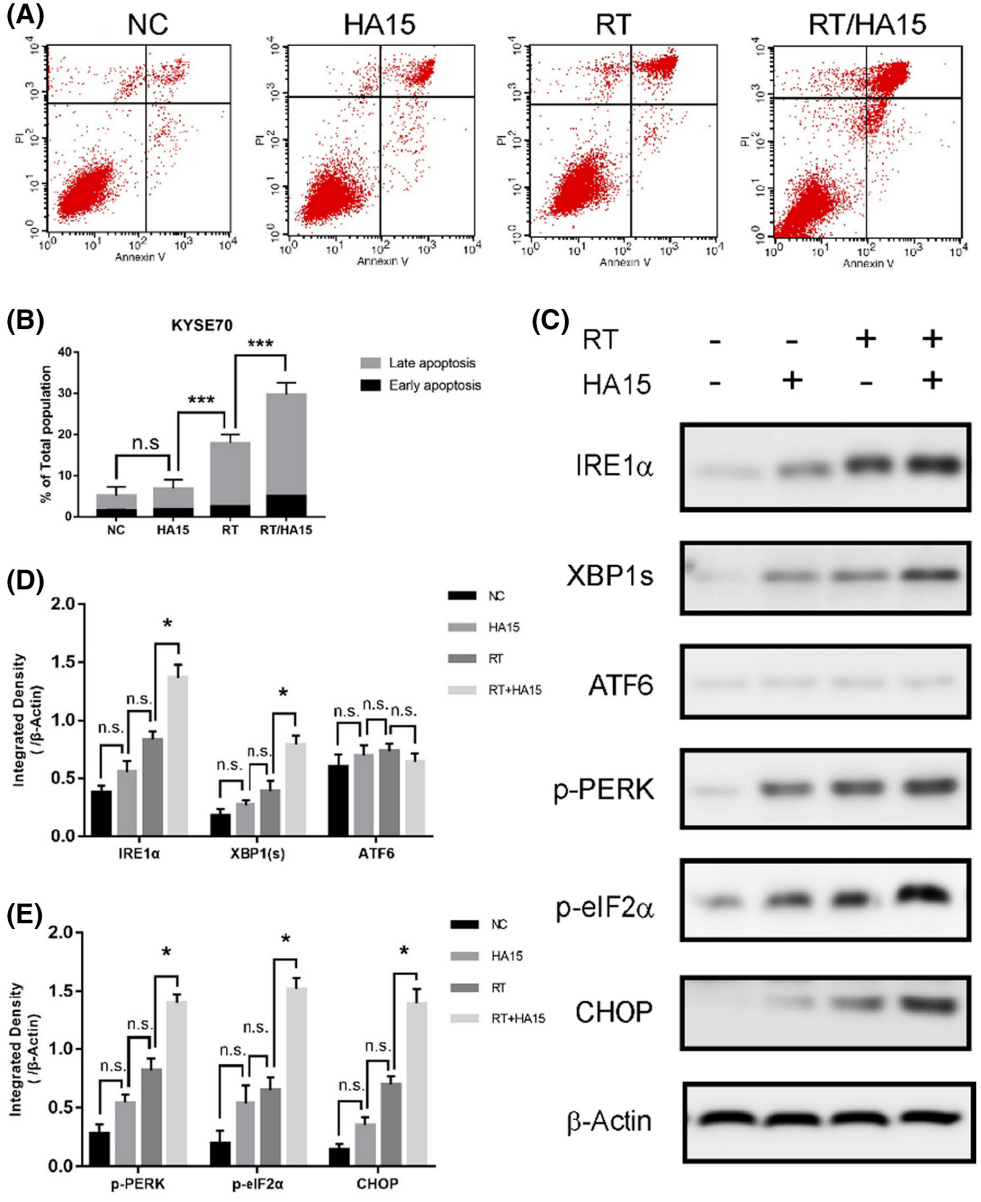


**FIGURE 5:**
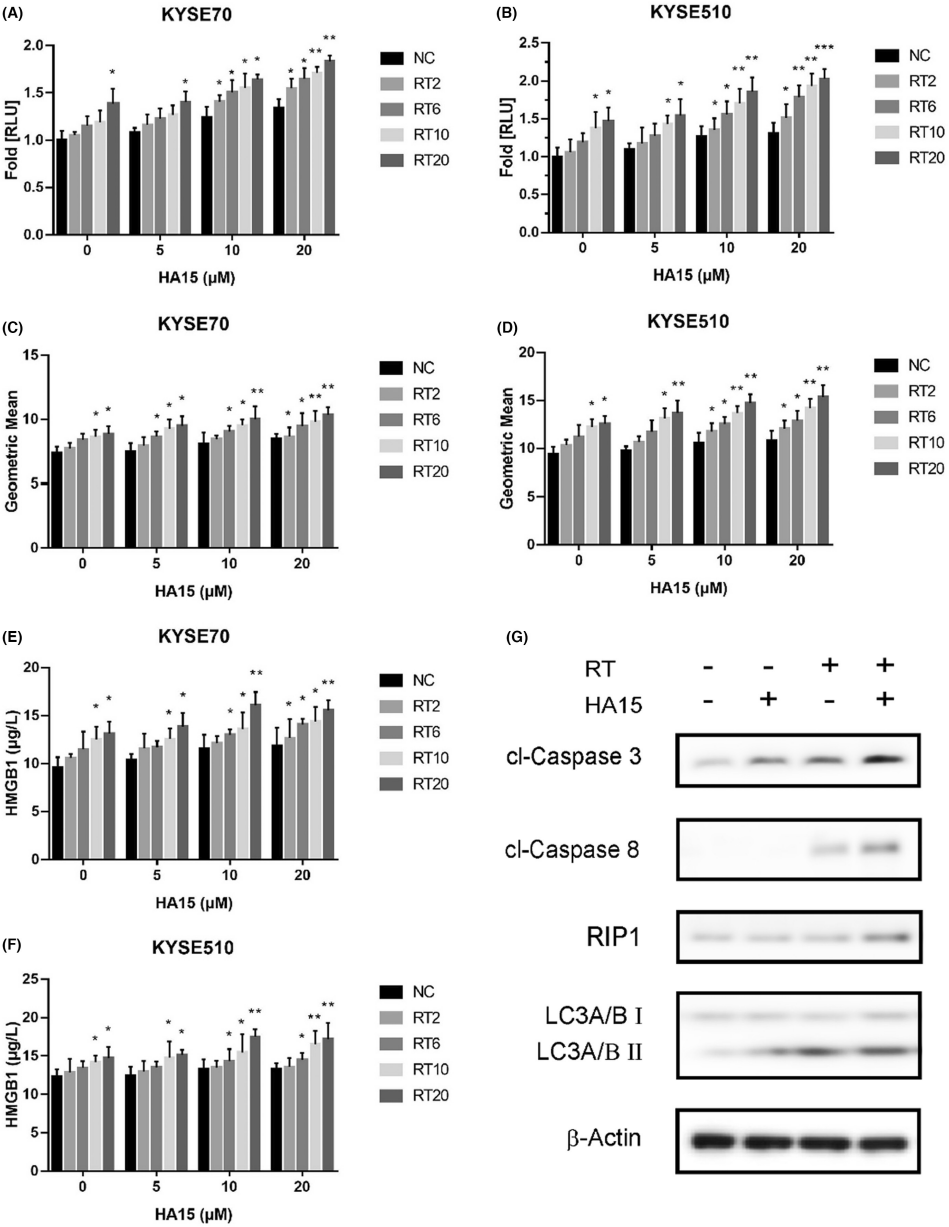


We apologize for this error.

